# A Space‐Time Conversion Vehicle for Programmed Multi‐Drugs Delivery into Pancreatic Tumor to Overcome Matrix and Reflux Barriers

**DOI:** 10.1002/advs.202200608

**Published:** 2022-05-04

**Authors:** Taotao Huo, Xiaoyi Zhang, Min Qian, Huifang Nie, Dong Liang, Chenteng Lin, Yafeng Yang, Wei Guo, Ulrich Lächelt, Rongqin Huang

**Affiliations:** ^1^ Department of Pharmaceutics School of Pharmacy Key Laboratory of Smart Drug Delivery Ministry of Education Fudan University Shanghai 201203 P. R. China; ^2^ Department of Pharmaceutical Sciences University of Vienna Vienna 1090 Austria

**Keywords:** covalent organic framework, multi‐drugs delivery, pancreatic carcinoma, programmed drug delivery

## Abstract

The numerous biological barriers, which limit pharmacotherapy of pancreatic carcinoma, including inadequate drug accumulation in the tumor environment, a dense extracellular matrix (ECM) and efficient drug‐efflux mechanisms, illustrate the requirement of multifunctional delivery systems to overcome the individual barriers at the right place at the right time. Herein, a space–time conversion vehicle based on covalent organic framework (COF)‐coated mesoporous silica nanospheres (MSN) with a sandwiched polyethyleneimine (PEI) layer (MPCP), is designed. The space‐specific drugs‐loaded vehicle (M_G_P_P_C_L_P) is obtained by separately incorporating a chemotherapeutic agent (gemcitabine, G) into the MSN core, a P glycoprotein inhibitor (LY 335979, P) into the PEI layer, and an extracellular matrix disruptor (losartan, L) into the COF shell. Thereafter, a programmed drug delivery is achieved via the ordered degradation from COF shell to MSN core. Sequential release of the individual drugs, synergized with a change of nanoparticle surface charge, contribute to an obvious extracellular matrix distraction, distinct drug efflux inhibition, and consequently enhance chemotherapeutic outcomes in pancreatic carcinoma. This MPCP‐based vehicle design suggests a robust space–time conversion strategy to achieve programmed multi‐drugs delivery and represents a new avenue to the treatment of pancreatic carcinoma by overcoming extracellular matrix and drug reflux barriers.

## Graphical abstract

A programmed drug delivery system (M_G_P_P_C_L_P) is obtained by spatially incorporating gemcitabine (G), P glycoprotein inhibitor (P), and losartan (L) into the MSN core, PEI layer, and COF shell, respectively, to achieve an extracellular matrix distraction, distinct drug efflux inhibition, and the enhanced chemotherapeutic outcomes for pancreatic carcinoma.

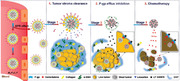

## 1 Introduction

Pancreatic carcinoma, which is one of the cancers with the poorest prognosis, has spurred the development of various drugs and therapeutic approaches.^[^ ^1^, ^2^, ^3^, ^4^ ^]^ Unfortunately, a dense extracellular matrix (ECM) in the pancreatic tumor microenvironment (TME), composed of cancer‐associated fibroblasts (CAF), collagen, inflammatory cells, and lymphatic vessels, severely impedes drug delivery into tumor cells due to physical barrier effects and associated high intracellular interstitial pressure.^[^ ^5^, ^6^, ^7^, ^8^, ^9^, ^10^ ^]^ Even worse, after penetrating the ECM, serious drug‐efflux caused by over‐expressed P glycoprotein (P‐gp) in tumor cells further limits the adequate drug accumulation within cancerous pancreatic cells.^[^ ^11^ ^]^ Therefore, various strategies have recently been explored to overcome the ECM and drug‐efflux barriers for pancreatic carcinoma drug delivery.^[^ ^12^, ^13^, ^14^, ^15^ ^]^ Among these, different pharmacological compounds, such as anti‐fibrotic drugs (losartan, tranilast, and pirfenidone),^[^ ^13^, ^14^, ^15^ ^]^ efflux inhibitor (curcumin),^[^ ^16^ ^]^ and chemotherapeutic drugs (paclitaxel and gemcitabine) ^[^ ^17^, ^18^ ^]^ have been used as simple and efficient treatments of the diverse barriers for pancreatic carcinoma therapy. However, in most cases, these drugs are usually simultaneously delivered or located in a homogeneous vehicle for simultaneous release. Since the critical barriers and target cancer cells are located at different positions within the delivery pathway, a simultaneous drug release at one place is suboptimal and does not take the spatial barrier distribution and onset times of different drugs into consideration. For example, the reversal of extracellular matrix should be done before chemotherapy and the efficient drug accumulation should occur after the suppression of tumor drug resistance. Meanwhile, the simultaneous drug release can even lead to the increasing multidrug resistance of tumor and the serious side effects to normal tissues.^[^ ^11^ ^]^ Therefore, a programmed drug release is urgently needed for tumor drug delivery, especially in case of stroma‐enriched and multidrug‐resistant pancreatic cancer.

Commonly, sequential medication using different nano‐drugs is proposed for programmed drug delivery. Nevertheless, this approach would generally increase the burden of medication and also not regulate well the action time especially in complicated physiopathological barriers.^[^ ^19^ ^]^ Therefore, simultaneous multi‐drugs delivery using a nanocarrier with programmed and sequential drug release is an optimal alternation. As known, space–time conversion, where the differences in time can be achieved via the different spatial distributions, is a potential strategy to realize the programmed drug delivery. The key point using this space–time conversion strategy for programmed drug delivery is the preparation of uniformed nanoparticles with non‐homogeneous compositions, where the drugs can be loaded into different compartments and gradually released due to the TME‐triggered degradation.

**Scheme 1:**
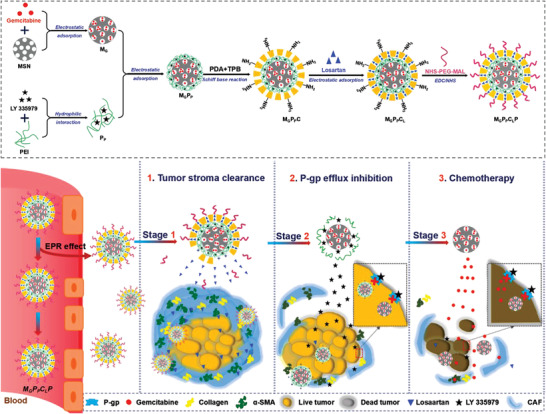
Schematic illustration of the preparation of M_G_P_P_C_L_P and the corresponding mechanism of programmed drug delivery for overcoming the matrix and reflux barriers in pancreatic carcinoma treatment.

In this work, a uniformed core–shell vehicle (MPCP) was prepared, where the covalent organic frameworks (COF) shell was coated on mesoporous silica nanospheres (MSN) via polyethyleneimine (PEI)‐mediated interface growth. Due to different compositions and properties of MSN, PEI and COF, a chemotherapeutic agent (gemcitabine, G), a P glycoprotein inhibitor (LY 335979, P), and an extracellular matrix disruptor (losartan, L) were separately incorporated into the MSN core, PEI layer, and COF shell of MPCP in sequence during the synthesis to construct a space‐specific drugs‐loaded nanoparticle (**Scheme** **1**). After PEGylation, the multi‐drugs loaded nanoparticle (M_G_P_P_C_L_P) was electroneutral, benefiting for the in vivo delivery to tumor areas. Upon exposure to the acid TME, the COF layer would be degraded and subsequently the PEI layer was swollen out, which bared the nanoparticle with positive charge for deep tumor penetration. More importantly, the gradual biodegradation from the COF shell to PEI layer was observed on the MPCP nanospheres, which promised a programmed drug delivery and release (Scheme 1). As a result, an obvious extracellular matrix disruption and P‐gp efflux inhibition were achieved to enhance the chemotherapy of pancreatic carcinoma. This space–time conversion strategy for programmed multi‐drugs delivery based on COF‐coated MSN with a sandwiched PEI layer paves a new way for stroma‐enriched pancreatic carcinoma treatment.

## 2 Result and Discussion

**Figure 1:**
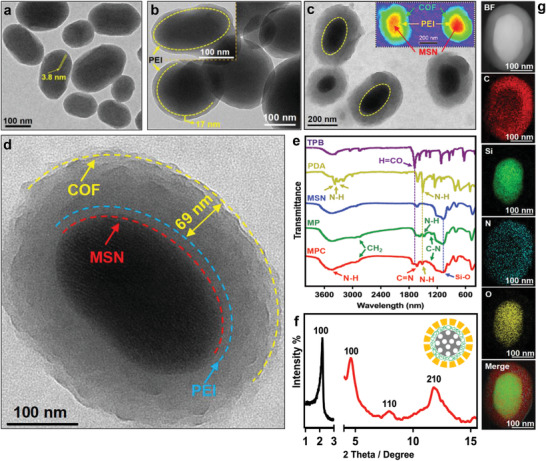
TEM images of a) MSN, b) MP (MSN@PEI), and c,d) MPC (MSN@PEI@COF). e) FT‐IR patterns of TPB (1,3,5‐benzenetricarboxaldehyde), PDA (p‐phenylenediamine), MSN, MP, and MPC. f) XRD pattern of MPC, insert: schematic structure of MPC. g) STEM of MPC and the corresponding EDX mappings of carbon, silica, nitrogen, and oxygen, respectively.

**Figure 2:**
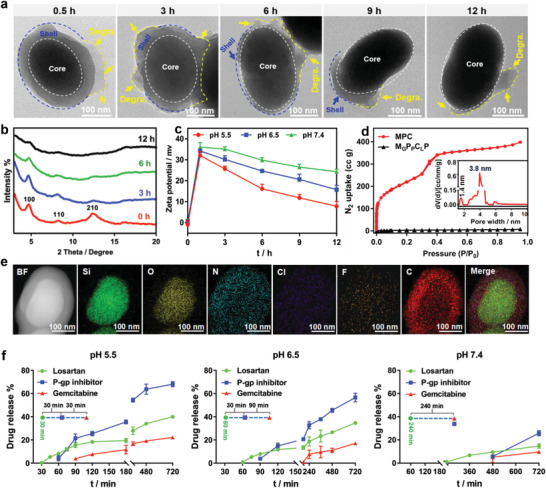
a) TEM images and b) XRD patterns of MPC incubated in pH 5.5 media for different time periods. Degra.: degradation. The white, blue, and yellow lines labeled MSN cores, PEI‐COF shells, and the degradation areas, respectively. c) Zeta potentials of MPC incubated in pH 5.5, 6.5, and 7.4 media for different time periods. d) Nitrogen adsorption–desorption isotherms and the corresponding pore size distribution curves (inset) of MPC and M_G_P_P_C_L_P. e) STEM images of M_G_P_P_C_L_P and its EDX mappings for silica, oxygen, nitrogen, chlorine, fluorine, carbon, and the merged mapping, respectively. f) The accumulated drug release profiles of M_G_P_P_C_L_P against PBS at pH 5.5, pH 6.5, and pH 7.4. Data are presented as mean ± SD (*n* = 3).

To construct the core–shell nanoparticle, MSN with oval shape (120–250 nm) and ordered ≈3.8 nm mesopores was first prepared (**Figure** **1**a and Figure S1, Supporting Information). This MSN had a negative zeta potential (−26 mV), and thus exhibited a mono‐dispersity as verified by a little larger DLS particle size than the TEM‐measured size (Figures S1–S3, Supporting Information). Then, positively charged PEI was coated on MSN (MSN@PEI, MP) via electrostatic interaction, which derived a uniformed polymer layer (about 15 ± 2 nm thickness) around MSN by the optimized synthesis according to the zeta potential (Figure 1b and Figure S2, Supporting Information). Although PEI coating resulted in a little aggregation of the nanoparticles, it provided a positively charged and aminated surface (Figure S2, Supporting Information). Thus, this facilitated the 1,3,5‐triformylbenzene (TPB) monomer linkage, whereafter the crystalline COF shell could grow on the surfaces of MP by the 1,4‐phenylenediamine (PDA) and TPB monomer‐mediated in‐situ Schiff reaction.^[^ ^17^ ^]^ As shown in Figure 1c,d, the as‐obtained nanoparticle exhibited an obvious core–shell architecture with typical COF coating on the PEI‐grafted MSN. The COF shell was 71 ± 7 nm and mainly composed of C and N elements, validating the Schiff polymerization reaction between TPB and PDA (Figure 1d,g, and Figure S4, Supporting Information).^[^ ^20^ ^]^ Furthermore, the obvious C—N vibration and N—H vibration emerged in the FTIR of MP, also confirmed the PEI coating. Especially, the decreased H═CO stretching vibration (1720 cm^−1^), N—H bending vibration (3380 cm^−1^), and the emerged C═N vibration (1670 cm^−1^) as compared with those of monomers (TPB, PDA) and MP confirmed the formation of COF via Schiff‐base polymerization reaction between aldehyde groups of TPB and amino groups of PDA (Figure 1e). This PEI coating and COF growth did not disrupt the ordered meso‐structure of MSN since the well‐kept (100) diffraction ^[^ ^21^ ^]^ in small angle XRD pattern and the obvious pore channels in the magnified TEM image (Figure 1e and Figure S5, Supporting Information). Correspondingly, obvious diffractions at 4.82°, 8.03°, and 12.52° indexed as (100), (110), and (210) planes of crystalline COF emerged in MPC,^[^ ^22^ ^]^ further demonstrated the ordered microporous polymer coating (Figure 1f). The dual‐ordered pores separated by a PEI layer suggested their potential drug storage, which would be site‐specific due to the hierarchical structure with diverse compositions. Moreover, owing to the elevated hydrolysis of Schiff‐base and the proton sponge effect of PEI under acidic environment, a gradual dissociation of the COF shell followed by the gradual fading out of PEI from the MSN core was observed on this special vehicle at a relatively low pH solution from the representative TEM images (**Figure** **2**a). Correspondingly, the gradually disappeared XRD diffractions also suggested the degradation of COF shell under acidic environment (Figure 2b). Notably, this nano‐vehicle could be easily PEGylated into nearly electroneutral surfaces via the reaction between the NHS groups of PEG and the residue amino groups of COF shell (Scheme 1), whereafter the acid‐triggered degradation induced an obvious change from electric neutrality to positive charge and then gradually decreased at especially the low pH media (Figure 2c). These confirmed the first dissociation of PEG‐grafted COF to expose the electropositive PEI and then the gradual PEI dissolution to bare MSN. These behaviors of MPCP facilitated the in vivo drug delivery since the electroneutral nanoparticle was beneficial for the increased blood circulation and TME‐triggered electro‐positivity which was useful for deep tumor penetration. More importantly, the unique biodegradation of MPCP could be utilized to regulate the drug release and mediate different cytotoxicities (Figure S6, Supporting Information), where the site‐specific drug loading would be converted to the programmed drug release.

To examine these, drugs with different functions including the chemotherapeutic agent (gemcitabine, G), the P glycoprotein inhibitor (LY 335979, P),^[^ ^23^ ^]^ and the extracellular matrix disruptor (losartan, L) ^[^ ^13^ ^]^ were incorporated into the MSN core, PEI layer, and COF shell via pore adsorption, hydrophilic, and electrostatic interactions, respectively (Scheme 1). The almost unchanged surface potentials (Figures S2 and S7, Supporting Information) and particle sizes (Figure S8, Supporting Information) as compared with the corresponding pristine nanoparticles at each‐step of incorporation suggested the drug loading into inner parts of each layer (Table S1, Supporting Information). Correspondingly, the drug loading rates were determined to be 10.5 ± 0.23% for gemcitabine, 1.04 ± 0.04% for LY 335979, and 9.64 ± 0.23% for losartan, respectively (Figure S9, Supporting Information). Therefore, this drug incorporation didn't apparently affect the COF growth as indicated by the reserved XRD pattern (Figure S10, Supporting Information) and the distinct core–shell nanoparticle (Figure S11, Supporting Information). Nevertheless, owing to the pore occupation by the drugs, the BET surface area and pore volumes of M_G_P_P_C_L_P measured by N_2_ adsorption–desorption isotherms were remarkably reduced (Figure 2d). Additionally, the additional F and Cl elements in M_G_P_P_C_L_P rather than MPCP also suggested the efficient drug loading into nanoparticles (Figure 2e and Figure S12, Supporting Information). Since each drug loading was carried out after each layer formation (behaving like the layer‐by‐layer assembly), these drug incorporations had a space‐specific location in this core–shell nano‐vehicle. Owing to the sequential dissociation of COF shell at acid conditions, this site‐specific drug loading in MPCP induced a synergistically pH‐responsive and time‐programmed release. As testified by the drug release curves in Figure 2f, all the three drugs had an elevated release ratio at relatively lower pH. Correspondingly, the drugs in the PEI layer (P, LY 335979) and particularly the MSN core (G, gemcitabine) had the obviously delayed release as compared with the drug (L, losartan) in the COF shell, which exhibited the drug location‐dependent time intervals at especially the acid environment. Therefore, this programmed release of different drugs delivered from the same nanoparticle was beneficial for pancreatic carcinoma treatments since it required different drugs to sequentially antagonize the diverse barriers.

**Figure 3:**
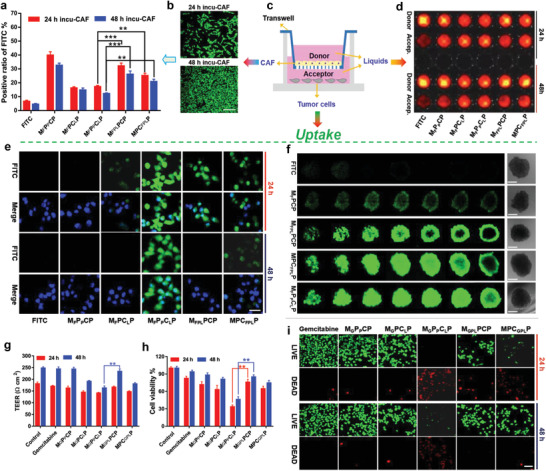
a) Flow cytometry analysis of different nanoparticles‐mediated FITC uptake by TGF‐*β* activated M 20 cell monolayers (CAF) incubated for 24 h or 48 h. Data are presented as mean ± SD (*n* = 3). Notes: **p < 0.01, ***p < 0.001. b) Densities of CAF incubated for different time periods. Scale bar: 100 µm. c) Schematic illustration of the transwell assay simulating the tumoral matrix‐barrier in vitro. CAF: TGF‐*β* activated M 20 cell monolayers; Donor: Dosing pool with CAF layer; Accep.: accepting pool with tumor cells. d) Fluorescence images of the supernatants in the donor and acceptor chambers after adding different formulations into the donor chambers for 6 h, where the CAF layers were incubated for 24 and 48 h, respectively. e) CLSM images of PANC‐1 cells in the acceptor chambers after adding different formulations in the donor chamber for 6 h, where the CAF layers were incubated for 24 and 48 h, respectively. Blue: DAPI; green: FITC. Scale bar = 20 µm. f) CLSM images of pancreatic tumor spheroids in the acceptor chambers after adding different formulations in the donor chambers for 10 h, where the CAF layers were incubated for 48 h. Green: FITC. Bar = 250 µm. g) TEERs of 24 or 48 h‐cultivated CAF after different treatments. Data are presented as mean ± SD (*n* = 3). Notes: **p < 0.01. h) CCK‐8 cell viabilities and i) live‐dead results of PANC‐1 cells in the acceptor chambers after adding different formulations in the donor chambers for 24 h, where the CAF were incubated for 24 and 48 h, respectively. Live: green; dead: red. Data are presented as mean ± SD (*n* = 3). Notes: **p < 0.01. Scale bar: 50 µm.

To investigate the performances of programmed multi‐drugs delivery vehicle (M_G_P_P_C_L_P) for pancreatic carcinoma treatment, a series of comparisons were carried out. Initially, a transwell‐chamber assay, where the TGF‐*β*‐activated M 20 cells (CAF) were incubated in the donor chamber for different time periods to imitate the pancreatic tumor external stroma with different densities ^[^ ^18^ ^]^ (**Figure** **3**b,c), and Panc‐1 cells or the corresponding tumor spheres were housed in the acceptor chamber to simulate pancreatic cancer cells with different growth statuses, was evaluated for the drugs loaded in different parts of the nanoparticle. Herein, FITC (F) was first used as the substitution for gemcitabine (G) in these nanoparticles to facilitate the examination of the uptake via fluorescence observation. As shown in Figure S13, Supporting Information, losartan‐loaded nanoparticles could decrease the TEER of CAF layer via their effect of matrix ablation, where the losartan loading in COF layer (M_F_PC_L_P, M_F_P_P_C_L_P, and MPC_FPL_P), especially the respective drug loading in different layers, showed the greatest impact on the 48 h‐incubated CAF. This suggested that the first released losartan without the interference of the simultaneously released other drugs could most effectively disrupt the extracellular matrix, particularly the compact one with 48 h incubation. In this situation, the FITC loaded in the MSN core of M_G_P_P_C_L_P had the minimum accumulation into CAF cells (Figure 3a). Since pure FITC could not be well taken up by CAF cells due to the absence of nanoparticle mediation (Figure 3a), M_F_P_P_C_L_P with the latest FITC release but the fewest FITC uptake could be attributed to its maximum CAF disruption by the released losartan in advance, allowing most FITC‐contained nanoparticles to cross it into the acceptor chamber (Figure 3d, and Figures S14 and S15, Supporting Information). From the fluorescence of supernatant liquids in the donor and acceptor chambers, it can also be concluded: 1) pure FITC could not well pass through the compact CAF layer and remain in the liquid of donor chamber due to the absence of nanoparticle mediation; 2) nanoparticles, especially the losartan‐contained ones, could elevate the transportation of FITC from donor chamber to acceptor chamber due to the simultaneous ablation effect of losartan disruptor to CAF layer besides the nanoparticle mediation; and 3) losartan that was loaded in the COF shell, particularly the one in the M_G_P_P_C_L_P, could achieve the most FITC transportation from donor chamber to acceptor chamber due to its foregoing release from the COF shell for matrix ablation and then the guaranteed FITC delivery across the matrix by the MSN core. Correspondingly, M_F_P_P_C_L_P also had the highest uptake by pancreatic cells in the acceptor chamber owing to its maximal matrix ablation (Figure 3e). Notably, although nano‐drugs with the single losartan incorporating in the COF shell had an efficient transfer from donor chamber to acceptor chamber, the P‐gp inhibitor‐absent one (M_F_PC_L_P) still didn't exhibit high tumor cell uptake and was even lower than the ones with three drugs co‐loaded in the MSN core or COF shell. This suggested the important role of LY 335979 incorporated in the PEI layer, which was released ahead of gemcitabine to inhibit the drug efflux. To further demonstrate the potentials of programmed drug delivery mediated by MPCP, tumor permeability of different nano‐drugs was studied on the Panc‐1 tumor spheres incubated in the acceptor chamber. As shown in Figure 3f, pure FITC could not penetrate into the tumor spheres due to the tumor interstitial pressure and multiple drug barriers. After loading in the MSN core, M_F_PCP had the same permeation into the tumor spheres possibly due to the electropositive nanoparticle delivery. Much more interestingly, losartan contained nanoparticles, especially the ones with losartan loading in COF shell (M_F_P_P_C_L_P and MPC_FPL_P), possessed a deeper drug penetration, confirming the optimal tumor matrix disruption of losartan via its release in advance (Figure S16, Supporting Information). Nevertheless, MPC_FPL_P‐mediated drug penetration was less than M_F_P_P_C_L_P. This could be attributed to the simultaneous release of all the three drugs in the COF shell, which made FITC apart from the vehicle. Subsequently, FITC could not well penetrate into the tumor due to the absence of the nanoparticle mediation despite the matrix disruption. And for M_FPL_PCP, losartan was loaded in MSN core and could not be effectively released to interfere with the external stroma. Therefore, FITC‐loaded nanoparticles could not well penetrate into tumor tissues. All these results displayed the programmed drug delivery by M_F_P_P_C_L_P for deep tumor penetration, which was beneficial for tumor therapy. Therefore, when gemcitabine was used as the chemotherapeutic agent, M_G_P_P_C_L_P also exhibited the most conspicuous tumor matrix disruption and accordingly the highest cytotoxicity to the Panc‐1 cells as compared with the other groups. These validated the programmed drug delivery by M_G_P_P_C_L_P for the most effective Panc‐1 cell treatment (Figure 3g–i).

**Figure 4:**
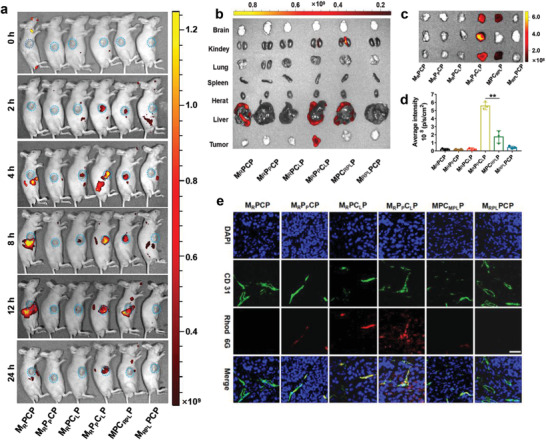
a) Images of subcutaneous pancreatic carcinoma‐bearing mice after the third intravenous injection of different drug formulations. Fluorescence: R, rhodamine 6G. b,c) Ex vivo fluorescence images of main organs and the three representative tumors excised from the tumor bearing mice at 24 h post‐injection of different drug formulations. d) Semi‐quantitative fluorescence intensities of tumors in Figure 4c. Data are presented as mean ± SD (*n* = 3). Notes: **p < 0.01. e) Immunofluorescence images of tumor sections from subcutaneous pancreatic carcinoma‐bearing mice at 24 h post‐injection of different drug formulations. Blue: nuclei; red: Rhodamine 6G; green: anti‐CD31 labeled blood vessels. Bar: 200 µm.

Next, the in vivo drug delivery of different nanoparticles was investigated via in vivo fluorescence imaging using rhodamine 6G (R) instead of gemcitabine based on subcutaneous tumor‐bearing mice. Owing to the prior ablation of tumor extracellular matrix via losartan and then the effective inhibition of drug efflux via LY 335979,^[^ ^24^ ^]^ intravenous injection of M_R_P_P_C_L_P induced the strongest fluorescence signals of rhodamine 6G in the tumor sites among all the groups (**Figure** **4**a and Figure S17, Supporting Information). Comparatively, M_R_PC_L_P could not inhibit the drug efflux due to the absence of P‐gp inhibitor, which thus had less rhodamine 6G accumulation in tumor than M_R_P_P_C_L_P despite its ability to disrupt the matrix. As for M_RPL_PCP and MPC_RPL_P, they suffered from either the weak matrix ablation due to delayed losartan release or the inadequate nanoparticle mediation due to the premature rhodamine 6G release. Therefore, tumor rhodamine 6G accumulation for M_RPL_PCP and MPC_RPL_P groups was also lower than that for M_R_PC_L_P. As for M_R_PCP, this nanosystem was mainly accumulated in the liver, presumably due to the intact physiopathological barriers and exclusion from the tumor (Figure 4a). In order to demonstrate these more clearly, the ex‐vivo organs at 24 h post‐injection were observed, which also revealed the maximal rhodamine 6G accumulation in tumor for the M_R_P_P_C_L_P group (Figure 4b–d). The superior drug delivery characteristics of M_R_P_P_C_L_P compared to other formulations verified the programmed drug release to overcome the multiple drug barriers. Additionally, the immunofluorescence study also revealed that more rhodamine 6G delivered by M_R_P_P_C_L_P accumulated into the tumor tissues rather than in blood vessels like the other formulations (Figure 4e). These results suggested that M_R_P_P_C_L_P, under the weak acid environment, could release the losartan, P‐gp inhibitor (LY 335979), and rhodamine 6G in sequence even if they were in the same nanoparticle. After the effective matrix cleanup by preferentially released losartan, the residual M_R_P_P_ with a relatively small size and positive charge could penetrate into the deeper regions of the tumor, which, being coupled with the following release of LY 335979 loaded in PEI layer, inhibited the drug efflux and improved the endocytosis effect. Thereafter, more rhodamine 6G in the MSN cores was released and accumulated in tumor cells.

**Figure 5:**
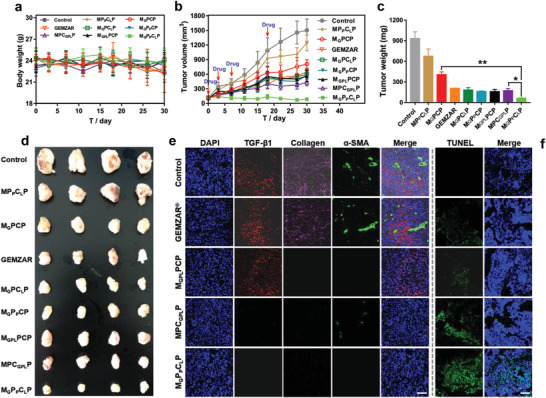
a) Average body weight and b) tumor growth curves of subcutaneous pancreatic carcinoma‐bearing mice after intravenous administration of different drug formulations (*n* = 8). c) Tumor weight and d) tumor optical images at 30th day post‐injection of different drug formulations. Data are presented as mean ± SD (*n* = 4). Notes: *p < 0.05, **p < 0.01. e) Immunofluorescence images of tumor sections from subcutaneous pancreatic carcinoma‐bearing mice after 15 days treatments. Blue: nuclei; purple: collagen; green: *α*‐SMA; red: TGF‐*β*1. Bar = 200 µm. f) Apoptosis results via TUNEL detection of the subcutaneous pancreatic carcinoma‐bearing mice after 15 days treatments. Blue: DAPI‐labeled cell nuclei; green: FITC‐labeled apoptotic cells. Bar = 200 µm.

Since the programmed drug delivery by M_G_P_P_C_L_P could overcome tumor matrix and drug efflux barriers and enhance the drug accumulation into tumor cells, it was considered to be a robust system for pancreatic carcinoma therapy, where thus the in vivo anti‐tumor activity was evaluated in pancreatic carcinoma‐bearing mice via intravenous injection of different formulations. During the experiment, none of the treatments caused an obvious change of body weight (**Figure** **5**a). As shown in Figure 5b,d, all drug‐loaded groups showed reduced tumor growth. Accordingly, at the 30th day after treatments, the tumor weight and tumor volumes of animals treated with drug‐loaded nanoparticles were smaller than those in the control group, suggesting the expected chemotherapeutic effects (Figure 5b–d). Notably, among all treatments, M_G_P_P_C_L_P exhibited the most pronounced effects and almost induced complete tumor growth inhibition. And TUNEL staining also detected the most obvious induction of apoptosis in the M_G_P_P_C_L_P group (Figure 5f). This could be attributed to the programmed multi‐drugs release with effective overcoming of the multiple drug barriers, which was superior to groups with the single barrier ablation (M_G_PC_L_P and M_G_P_P_CP) or the only chemotherapy (M_G_PCP and GEMZAR), and also escaped from the increased body clearance caused by the absonant drug release (M_GPL_PCP and MPC_GPL_P). This point was further verified by the immunofluorescence assay, where the tumor stroma including the collagen, *α*‐SMA, and TGF‐*β*1 showed a similar level between control and GEMZAR, indicating that gemcitabine alone could not effectively inhibit the tumor stroma.^[^ ^13^, ^25^ ^]^ (Figure 5e). Comparatively, losartan‐contained formulations, especially loaded in the COF layer, induced obvious stroma clearance with the decreased collagen, *α*‐SMA, and TGF‐*β*1 signals due to the preferential losartan release. Notably, the stroma clearance for MPC_GPL_P was still a bit weaker than that for M_G_P_P_C_L_P, which might be attributed to the antagonism effects with the decreased stroma clearance ability of the simultaneous release of three drugs. Therefore, the programmed multi‐drugs delivery via M_G_P_P_C_L_P had advanced therapeutic outcomes of pancreatic carcinoma.

**Figure 6:**
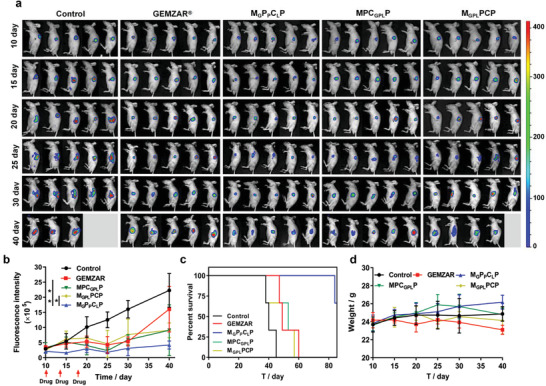
a) Bioluminescence images, b) fluorescence intensity in the tumors, c) Kaplan–Meier survival curves, and d) body weight curves of orthotopic pancreatic carcinoma‐bearing mice after intravenous administration of different formulations. Data are presented as mean ± SD (*n* = 5). Notes: *p < 0.05, **p < 0.01.

Finally, to confirm the therapeutic effects of M_G_P_P_C_L_P for pancreatic carcinoma treatment, these different formulations were examined on the orthotopic pancreatic tumor‐bearing mice constructed by pancreatic injection of luc‐modified Panc‐1 cells with bioluminescence. As shown in **Figure** **6**a,b, the saline‐treated group had a rapid tumor growth during the experimental period, while gemcitabine obviously inhibited the tumor growth. Comparatively, the combined multi‐drugs treatments, especially the M_G_P_P_C_L_P treatment with programmed drug release, mediated a much more significant growth inhibition of orthotopic pancreatic tumor, where the M_G_P_P_C_L_P therapy produced the most prolonged median survival time of 84 days among all these different treatments (Figure 6c). Additionally, the therapy of the orthotopic pancreatic tumor using these different nano‐formulations didn't bring any obvious change in the body weight of mice (Figure 6d). Conclusively, M_G_P_P_C_L_P would be an efficient multi‐drugs delivery vehicle for potential tumor therapy by overcoming the complicate barriers.

## 3 Conclusion

In summary, a space–time conversion vehicle based on COF‐coated MSN with a sandwiched PEI layer (MPCP), was synthesized via PEI‐mediated interface growth. Attributing to the diverse compositions and pore structures, a chemotherapeutic agent (gemcitabine, G), a P glycoprotein inhibitor (LY 335979, P), and an extracellular matrix disruptor (losartan, L) were separately incorporated into the MSN core, PEI layer, and COF shell of MPCP in sequence during the synthesis, obtaining a space‐specific drug‐loaded nanoparticle (M_G_P_P_C_L_P). Whereas, upon exposure to the acid TME, the COF shell, and PEI layer of the nanoparticles could be successfully biodegraded or swollen out, which not only bared the nanoparticles with positive charge for deep tumor penetration, but also contributed to a programmed drug release. Benefiting from these, the obvious extracellular matrix ablation and drug efflux inhibition were achieved, which thus promised an enhanced chemotherapeutic outcome of pancreatic carcinoma in vivo. This work provided a robust drug delivery vehicle for efficient pancreatic carcinoma treatment, suggesting the space–time conversion strategy for programmed drug delivery to overcome the physiopathological barriers of tumor.

## Conflict of Interest

The authors declare no conflict of interest.

## Supporting information

Supporting Information

## Data Availability

The data that support the findings of this study are available on request from the corresponding author. The data are not publicly available due to privacy or ethical restrictions.
