# Supplementary material for: A Space‐Time Conversion Vehicle for Programmed Multi‐Drugs Delivery into Pancreatic Tumor to Overcome Matrix and Reflux Barriers
Source: Adv Sci (Weinh). 2022 May 4;9(20):2200608. doi: 10.1002/advs.202200608 (PMC9284157; doi:10.1002/advs.202200608)
Supplement: Supplementary file 1 — Supporting Information [file ADVS-9-2200608-s001.pdf]

ADVANCED SCIENCE
Open Access

Supporting Information

for *Adv. Sci.*, DOI 10.1002/advs.202200608

A Space-Time Conversion Vehicle for Programmed Multi-Drugs Delivery into Pancreatic Tumor to Overcome Matrix and Reflux Barriers

*Taotao Huo, Xiaoyi Zhang, Min Qian, Huifang Nie, Dong Liang, Chenteng Lin, Yafeng Yang, Wei Guo, Ulrich Lächelt and Rongqin Huang**

A Space-Time Conversion Vehicle for Programmed Multi-drugs Delivery into Pancreatic Tumor to Overcome Matrix and Reflux Barriers

Taotao Huo [a], Xiaoyi Zhang [a], Min Qian [a], Huifang Nie [a], Dong Liang [a], Chenteng Lin [a], Yafeng Yang [a], Wei Guo [a], Ulrich Lächelt [b], Rongqin Huang [a,*]

[a] Department of Pharmaceutics, School of Pharmacy, Key Laboratory of Smart Drug Delivery, Ministry of Education, Fudan University, Shanghai 201203, China

[b] Department of Pharmaceutical Sciences, University of Vienna, Vienna, Austria

Materials: Tetraethyl orthosilicate (TEOS) and 1,3,5-triformylbenzene (TPB) were purchased from Yien Chemical Technology Co., Ltd. (Shanghai, China). Hexadecyltrimethylammonium bromide (CTAB) was acquired from Shengwei Chemical Raw Materials Co., Ltd. (Shanghai, China). 1,4-phenylenediamine (PDA) and dioxane were obtained from Aladdin (Shanghai, China). Glacial acetic acid and butylene oxide were purchased from Jinan Mengqiao Chemical Co., Ltd. (Shandong, China). Polyethylene glycol derivative (NHS-PEG-MAL, MW 3500) was bought from Jenkem Technology (Beijing, China). Fluorescein isothiocyanate isomer (FITC, F), gemcitabine (G) and losartan (L) were bought from Aladdin (Shanghai, China). Cell Counting Kit-8 (CCK-8) was bought from Beyotime Biotechnology (Shanghai, China). Gemcitabine hydrochloride for injection (GEMZAR®) was purchased from Vianex S.A.-Plant C (Greece). Polyethyleneimine (PEI_{600}) was bought from Alfa Aesar. Zosuquidar trihydrochloride (P-gp inhibitors, LY 335979, P) and Rhodamine 6G (R) were bought from Sigma (Saint Louis, USA). TUNEL Apoptosis Detection Kits (FITC-labeled) were bought from Shanghai Yisheng Biotechnology Co., Ltd. (Shanghai, China). Fetal bovine serum (FBS) was obtained from Biochannel (Nanjing, China). Dulbecco's modified Eagle's medium (DMEM), Tyrisin and L-glutamine were obtained from Gibco. Transforming growth factor-β (TGF-β) was obtained from AbMole. (California, USA). Live/Dead cell double staining kits were obtained from Sigma (St. Louis, USA).

Synthesis of mesoporous silica nanosphere (MSN): 100 mg cetyltrimethylammonium bromide was dissolved in sodium hydroxide solution (0.583 mg/mL) by magnetic stirring and then 80 °C oil bath heating. When the temperature was stable, 500 μL tetraethyl orthosilicate was slowly added with a pipette. After magnetic stirring for 2 h, the raw MSN was washed with absolute ethyl alcohol for several times to remove residual surfactant from the MSN, and the pure product was lyophilized.

Synthesis of MSN@PEI (MP): 30 mg/mL PEI and 5 mg/mL MSN suspension were mixed with magnetic stirring for 4 h. After that, free PEI was removed by centrifugation (10000 rpm/5min) and the precipitate (about 50 mg) were resuspended in 1,4-Dioxane for following synthesis.

Synthesis of heterogeneous nucleus: Triformylbenzene (5 mg) and MSN@PEI (50 mg) were mixed in 1 mL dioxane by magnetic stirring. After that, 20 μL phenylenediamine-containing acetic acid were added. And then, the reaction mixture was reduced to 2 mL with dioxane. After reaction, the suspension was used immediately for the next preparation step.

Synthesis of MSN@PEI@COF (MPC): 10 mg phenylenediamine and 12 mg triformylbenzene were mixed in 1 mL MSN-dioxane heterogeneous nucleus media. After vortexing, 80 μL acetic acid was added with a pipette. And then, 2 mL reaction mixture was added into a round-bottom flask with magnetic stirring for 3 h in which the temperature was maintained at 120 ℃ with an oil bath. The middle products of MPC were collected and the qualified MPC were acquired by vacuum drying at 70 ℃.

Preparation of $M_GP_PC_LP$: Firstly, MSN was mixed with gemcitabine at different concentrations in ultrapure water (pH 7.4) while PEI was mixed with LY 335979 at different concentrations in ultrapure water (pH 7.4) by magnetic stirring for 6 h, respectively. Free drugs in solution were removed by centrifugation. Secondly, gemcitabine loaded MSN (M_G) was coated by LY 335979 loaded PEI (P_P) through electrostatic interaction with a mass ratio of 1:6. After that, the precipitates (M_GP_P) were redispersed in dioxane and COF was prepared according to the procedures described above (**Scheme 1**). For package of losartan, the COF modified M_GP_P was mixed with losartan at different concentrations in ultrapure water (pH 7.4) by stirring for 4 h. After the free gemcitabine was removed, PEGylation was carried out in $M_GP_PC_L$ by functionalized PEG_{3500} at the ratio 1:1 (w/w). After centrifugation (12000 rpm/10 min), the precipitates (PEGylation $M_GP_PC_L$, $M_GP_PC_LP$) were collected by centrifugation for further use. The encapsulation efficiency (EE%) and drug loading rate (DL%) of $M_GP_PC_LP$ were determined by RRLC-MS/MS (SCIEX QTRAP® 5500).

Preparation of other MPCP formulations: For the preparation of M_GPCP, M_GP_PCP, M_GPC_LP and MP_PC_LP, the synthesis processes were consistent with that of $M_GP_PC_LP$, except that the corresponding drug loading nanolayer was replaced by a blank carrier. And for the preparation of $M_{GPL}PCP$, MSN was firstly mixed with gemcitabine (G), LY 335979 (P) and losartan (L) at the same concentration in ultrapure water (pH 7.4) by magnetic stirring for 6 h. After removing unpacked drugs by centrifugation, drugs-loaded MSN (M_{GPL}) was coated by PEI with a mass ratio

of 1:6. After that, $M_{GPL}P$ was redissolved in dioxane and COF was prepared according to the procedures described in **Scheme 1** without losartan loading. For the preparation of $MPC_{GPL}P$, blank MPC was prepared according to the process described in **Scheme 1**, and then MPC was mixed with gemcitabine (G), LY 335979 (P) and losartan (L) at the same concentration in ultrapure water (pH 7.4) by magnetic stirring for 6 h. After removing unpacked drugs by centrifugation, MPC_{GPL} was coated by PEG. And for the study of fluorescence imaging, MPCP formulations were prepared as described in **Scheme 1** in which FITC and rhodamine 6G substituted gemcitabine for the preparation of nanoparticles.

Characterizations: The particle size and zeta potentials of MSN, MSN@PEI, MPC and MPCP with or without drug loading were measured by Malvern potential/particle analyzer in which the concentration of the nanoparticles in pure water was 20 μg/mL. The morphology and elemental composition distribution of MPC and corresponding acid responsive degradation products were observed using a JEOL-2100F transmission electron microscope (TEM, Japan). XRD patterns of MPCP, its intermediate products and corresponding acid responsive degradation products were obtained with a Rigaku Ultima IV diffractometer (X'Pert PRO MPD). Infrared spectra (IR) of MSN, MSN@PEI, MPC and MPCP were obtained with a Thermo Nicolet AVATAR 360FT-IR using KBr pellet method. N_2 adsorption desorption isotherms and pore size distribution curves of MPCP with or without drug loading were measured using Tristar 3000 systems. And the degradation of COF shell under pH 5.5 was studied by XRD patterns. For study of drug release behavior, $M_GP_PC_LP$ was package into the 3500 Da dialysis units and suspended in 25 mL media with different pH values under magnetic stirring at 37 °C, respectively. At the pre-specified points, the dissolution media (200 μL) were collected and fresh and pre-heated release medium with equal volume was added. The released losartan, LY 335979 and gemcitabine were measured by Rapid Resolution Liquid Chromatography tandem-Mass Spectrometry (RRLC-MS/MS, SCIEX QTRAP® 5500).

RRLC-MS/MS: Chromatographic condition was performed on an Agilent Eclipse Plus C_{18} (50 × 2.1 mm, 1.8 μm). The column temperature was 30 °C and mobile phase included 0.01 moL/L H_3PO_4 (pH = 7.0) and acetonitrile with a linear gradient of 15 ~ 70% acetonitrile. The ESI-MS spectra were performed on a triple quadrupole mass spectrometry (SCIEX QTRAP® 5500) in multiple reaction monitoring (MRM) mode with an electrospray ion source. For positive ion mode, MRM transitions were set at m/z 264.7→112.1 for gemcitabine, m/z 528.8→240.8 for LY 335979 and 423.1→376.9 for losartan. The ionspray voltage was 5.5 kV and the nebuliser gas temperature was set at 550 °C with a flow of 7 L/min.

Cell lines and animals: Human pancreatic carcinoma cell lines (Panc-1) and human embryonic fibroblast cell lines

(M 20) were purchased from the Chinese Academy of Science cells Bank (Shanghai, China). Luc modified Panc-1 cells were acquired from Hanbio Biotechnology (Zhejiang, China). All the cells maintained in DMEM with 10% FBS, 1% penicillin, L-glutamine and streptomycin were cultured with 5% CO_2 at 37 °C. Nude mice (~ 20 g body weight) were obtained from the Department of Experimental Animals, Fudan University. All studies involving animals were carried out under the guidelines evaluated and approved by the ethics committee of Fudan University.

Cytotoxicity Analysis: Panc-1 cell and M 20 cell were seeded in 96 well plates, respectively. After adherent growth for 24 h at 37 °C, complete media containing different MPCP were added into cell modified well plates respectively to co-incubate for another 24 h. After that, the cell viability test was carried out by CCK-8 test in which the absorbance (A450) was measured with a microplate reader and the cell survival rate was calculated.

Study of cellular uptake of MPCP formulations against the extracellular matrix interference: Transwell chamber (donor chamber) were used for constructing a pancreatic cancer model in vitro. TGF-β activated M 20 cells were cultured in the transwell chamber with a density of 3×10^4 cells/well as cancer-associated fibroblasts (CAF) monolayers. After incubation for 24 h and 48 h, respectively, donor chambers were moved to 24-well plate with or without Panc-1 modification (acceptor chamber). And then the media contained different formulations (200 μg/mL FITC-equiv) were added to CAF monolayers modified transwell chamber. After intervention for 6 h, the media in donor chamber and Panc-1 free acceptor chamber were collected for fluorescence imaging. The FITC fluorescent intensity of M-20 in the donor chamber was evaluated by flow cytometry and the FITC fluorescent intensity of Panc-1 cells in the acceptor chamber were acquired by confocal lase scanning microscopy. The transepithelial electrical resistance (TEER) values of CAF monolayer were recorded by Millicell ERS electrical resistance system during the experiments. For cytotoxicity study, the media contained different formulations (equivalent to 200 μg/mL gemcitabine) were added to CAF monolayers. And after 24 h co-incubation, the cell viability was carried out by CCK-8 kits in which the absorbance (A450) was measured with a microplate reader and the cell survival rate was calculated. Meanwhile, the qualitative assessment of cell viability was carried out by live/dead cell imaging kits.

The tumor spheroids penetration of MPCP formulations against extracellular matrix interference: For 3D tumor spheroids preparation, Panc-1 cells (5×10^3/300 mL per well) were seeded in 48 well plates which were modified with 3% (w/v) low melting point agarose and complete media was changed every other day. After incubation for 5~7 days, the uniform tumor spheroids with about 500 μm diameter were selected. And the CAF monolayers were established as described above. After 48 h incubation with complete media, donor chambers were

moved to Panc-1 tumor spheroids modified 24 pore plate (acceptor chamber). And the complete media contained different formulations (equivalent to 200 µg/mL FITC) were added to donor. And after co-incubation for 10 h, the penetration of FITC-loaded COF nanocarriers in Panc-1 tumor spheroids were acquired by confocal laser scanning microscopy. The TEER values of CAF monolayers were recorded by a Millicell ERS electrical resistance system during the experiments.

In vivo imaging: To better mimic the composition of the stroma-enriched pancreatic cancer tumor, the subcutaneous pancreatic carcinoma model was established by injection of the mixture of 100 µL Panc-1 cells (2×10^6) and M 20 cells (0.5×10^6), a human fibroblast cell line, into the right back of nude mice. And the mice with tumor volume growing to about 150 mm^3 were used for studies. And to overcome the extracellular matrix barriers and achieve more accumulation of rhodamine 6G in tumor tissues, MPCP formulations were administered with 3 times through the tail vein over 7 days (25 mg/kg rhodamine 6G-equiv.). After the last injection, the mice were imaged with an IVIS Spectrum in-vivo imaging system (Caliper, USA) (ex: 520 nm) at preset time points. And the mice were kept under 10% chloral hydrate mediated anesthesia during the imaging study. At the 24 h post injection, the mice of each treatment group were sacrificed and the main organs including tumor tissues were collected and imaged. Meanwhile, the tumors were extracted to prepare frozen sections. The nucleus and tumor vessels were stained with DAPI and anti-CD31 antibody, respectively. The fluorescence co-location images were obtained by CLSM.

The anti-pancreatic cancer effect: The subcutaneous tumor model was established as described above. And treatments were carried out when tumor volume reached about 100 mm^3. The mice were weighed and randomly divided into several groups (8 mice per group) and the treatments (25 mg/kg gemcitabine-equiv) were carried out according to the designed regimen. For all groups, the tumor volume, tumor weight and the body weight were monitored. At the 15th day after administration, the mice of each group were sacrificed and the tumors were embedded by OTC tissue freezing medium to prepare the frozen sections. FITC-labeled TUNEL apoptosis detection kit was used for apoptotic cells detection. And for study of extracellular matrix components with different treatments, anti-CD31 was used for the blood vessels staining, anti-LF-68 was used for collagen I staining, anti-C6198 was used for aSMA staining and anti G122A was used for active TGF-b1 staining, respectively. After incubation for 12 h at 4 °C, tissue slices were further labeled with the corresponding secondary antibody. The fluorescence co-location images were obtained by confocal laser scanning microscope and the nucleus were labeled with DAPI. The orthotopic pancreatic carcinoma model was carried out by injection of 2×10^6 Luc-Panc-1 cells

into the pancreas head of nude mice. After 10 days, mice were injected with different formulations (25 mg/kg gemcitabine-equiv.) three times within 7 days. Bioluminescence images of mice were captured using an IVIS Spectrum in-vivo imaging system (Caliper, USA) at the 0th, 5th, 10th, 15th, 20th and 30th day after administration. Meanwhile, the overall survival and body weight of mice were monitored.

Statistical Analysis: The comparisons between data were executed by Student's t-test and ANOVA. All graphics were prepared by using GraphPad Prism 7 and all results were reported as means ± standard deviation (SD). Notes: *P < 0.05, **p < 0.01 and ***P < 0.001.

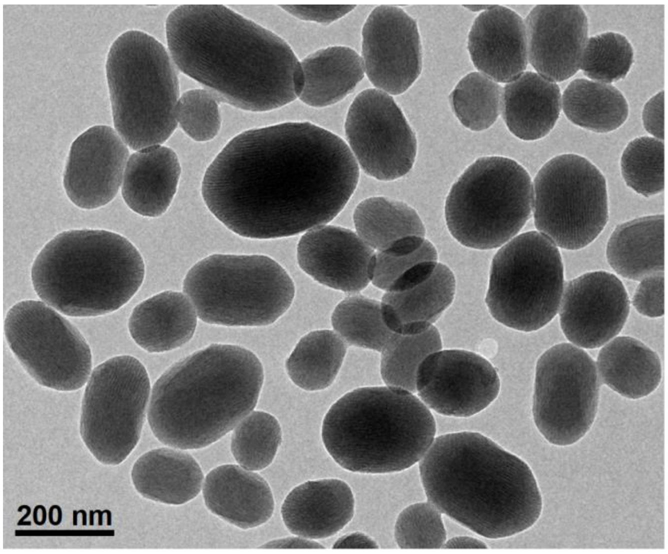

Figure S1 TEM image of MSN.

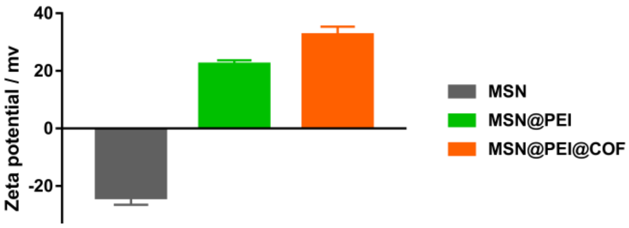

Figure S2 Zeta potential of MSN, MSN@PEI (MP) and MSN@PEI@COF (MPC). Data are presented as mean ± SD (n = 6).

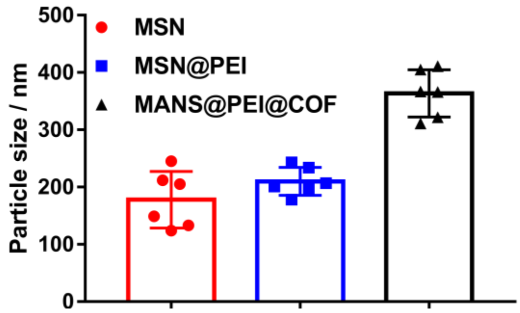

Figure S3 Particle size of MSN, MSN@PEI (MP) and MSN@PEI@COF (MPC). Data are presented as mean ± SD (n = 6).

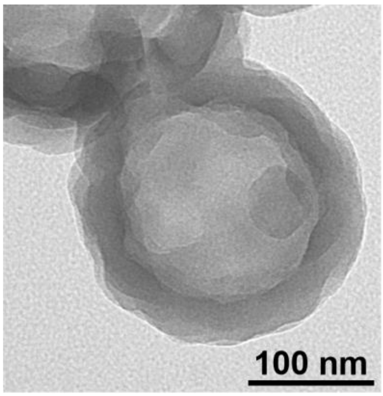

Figure S4 TEM image of hollow COF nanospheres.

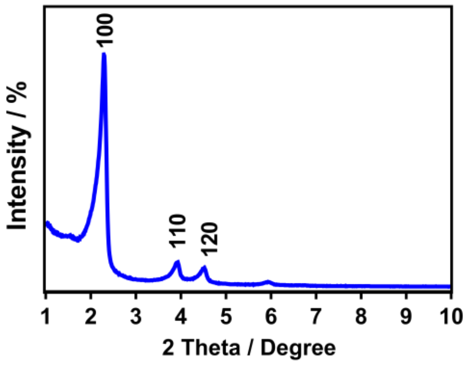

Figure S5 XRD pattern of MSN.

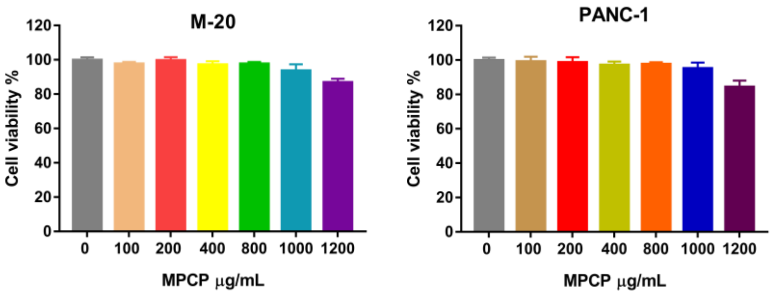

Figure S6 Cytotoxicity assay of MPCP on M-20 cells and Panc-1 cells by CCK-8 test. Data are presented as mean ± SD (n = 4).

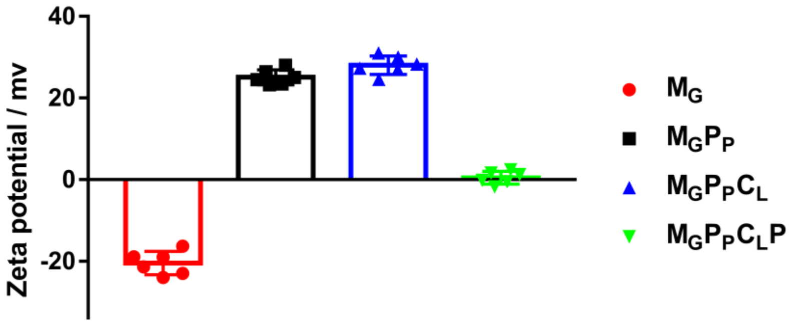

Figure S7 Zeta potentials of M_G, M_GP_P, $M_GP_PC_L$ and $M_GP_PC_LP$. Data are presented as mean ± SD (n = 6).

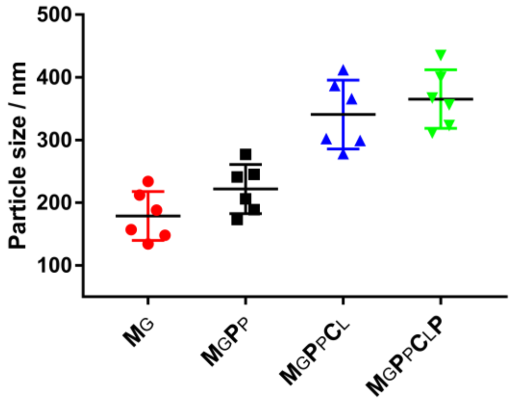

Figure S8 Particle sizes of M_G, M_GP_P, $M_GP_PC_L$ and $M_GP_PC_LP$. Data are presented as mean ± SD (n = 6).

Table S1 Physicochemical properties of nanocarriers and drugs.

Carrier				Drug				
**Name**	**Particel Size (nm)**	**Zeta potential (mv)**	**Pore size (nm)**	**Name**	**Molecular weight**	**Solubility (mg/mL)**	**Zeta potential (mv)**	**Structural formular**
**MSN yolk**	110 ± 9	- 25.6	3.8	**Gemcitabine hydrochloride**	299.66	≥ 10	30.23	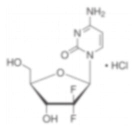
**PEI layer**	15 ± 2	23		**LY 335979**	637	≥ 5	20.37	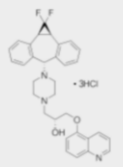
**COF shell**	70 ± 8	32	1.4	**Losartan**	461	> 50	-34.2	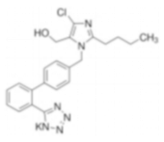

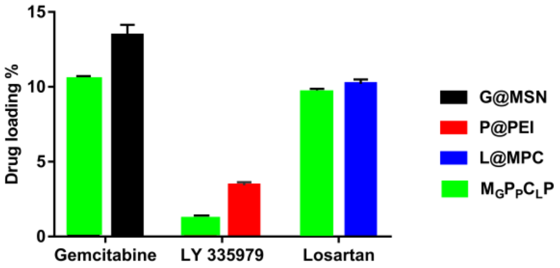

Figure S9 The drug loading efficiency of gemcitabine-loaded MSN (G@MSN), LY 335979-loaded PEI (P@PEI), losartan-loaded COF (L@COF) and multi-drugs loaded MPCP ($M_GP_PC_LP$). Data are presented as mean ± SD (n = 3).

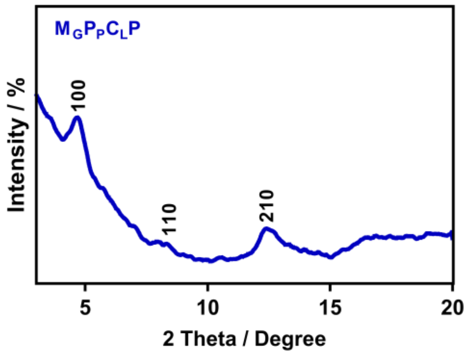

Figure S10 XRD pattern of $M_GP_PC_LP$.

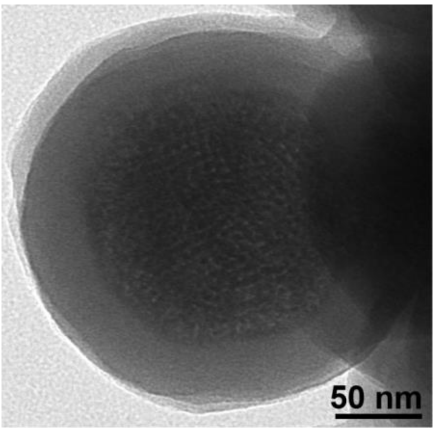

Figure S11 TEM images of $M_GP_PC_LP$.

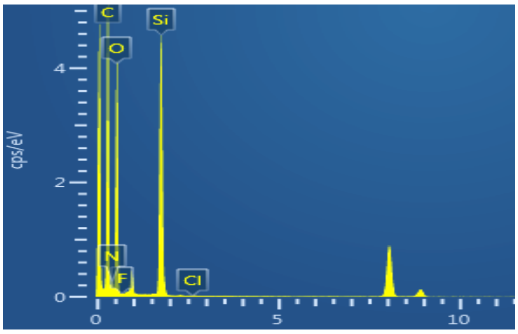

Figure S12 EDX pattern of $M_GP_PC_LP$.

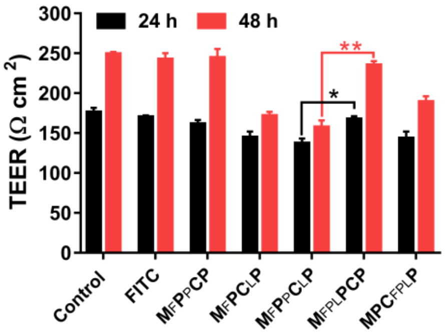

Figure S13 The TEER of 24 h and 48 h-cultivated CAF monolayer after different treatments. Data are presented as mean ± SD (n = 3), Notes: *p < 0.05, **P<0.01.

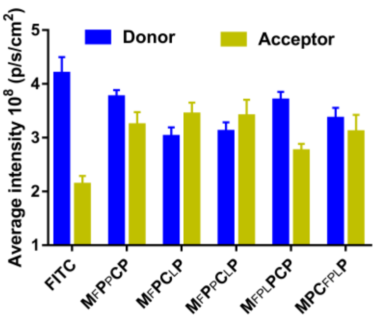

Figure S14 Average fluorescence (FITC) intensities of the liquids in the donor and acceptor chambers after adding different formulations into the donor chambers for 6 h, where the CAF layers were cultivated for 24 h. Data are presented as mean ± SD (n = 3).

.

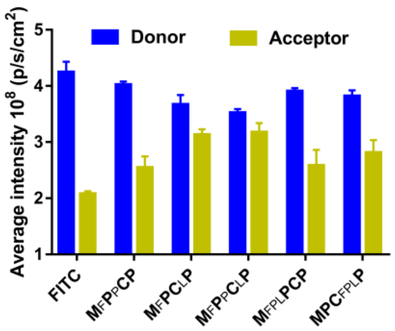

Figure S15 Average fluorescence (FITC) intensities of the liquids in the donor and acceptor chambers after adding different formulations into the donor chambers for 6 h, where the CAF layers were cultivated for 48 h. Data are presented as mean ± SD (n = 3).

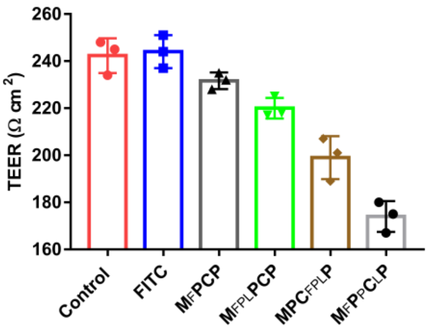

Figure S16 The TEER of CAF monolayers with 48 h incubation after different treatments. Data are presented as mean ± SD (n = 3).

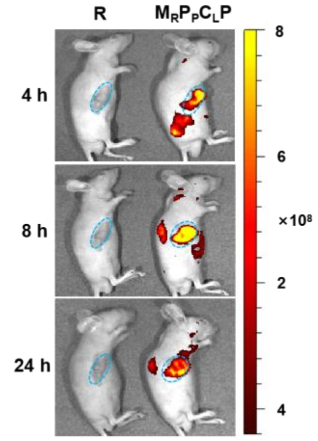

Figure S17 In vivo fluorescence imaging of subcutaneous pancreatic carcinoma-bearing mice after intravenous injection of $M_RP_PC_LP$ and free rhodamine 6G (R) at various time points.
